# German-Wide Analysis of the Prevalence and the Propagation Factors of the Zoonotic Dermatophyte *Trichophyton benhamiae*

**DOI:** 10.3390/jof6030161

**Published:** 2020-09-03

**Authors:** Max Berlin, Christiane Kupsch, Lea Ritter, Benjamin Stoelcker, Anton Heusinger, Yvonne Gräser

**Affiliations:** 1 Institute of Microbiology and Immunology, Humboldt-Universität zu Berlin and Berlin Institute of Health, corporate member of Freie Universität Berlin, Universitätsmedizin Berlin, 12203 Berlin, Germany; m.berlin.biotech@gmail.com (M.B.); christiane.kupsch@charite.de (C.K.); lea-ritter-2@web.de (L.R.); 2 SYNLAB Vet GmbH, 86156 Augsburg, Germany; Benjamin.Stoelcker@synlab.com; 3 LABOKLIN GmbH & Co. KG, 97688 Bad Kissingen, Germany; heusinger@laboklin.com

**Keywords:** dermatophyte, *Trichophyton benhamiae*, epidemiology, prevalence, guinea pig, emerging pathogen, breeding, pets

## Abstract

For about 10 years, a new variant of the pathogen *Trichophyton* (*T.*) *benhamiae* has appeared in Germany, characterized by a previously unobserved culture phenotype with a strong yellow reverse. A few studies suggest that this new variety is now the most common zoophilic dermatophyte in Germany. The guinea pig is the main carrier. Exact prevalence measurements are not yet available. Thus, the aim of our ongoing study was to collect data on the frequency and geographic distribution of the pathogen and its phenotypes (white and yellow) in humans and guinea pigs throughout Germany. Our former studies have already shown that animals from large breeding farms are particularly heavily affected. In contrast to this, 21 small, private breedings were sampled and husbandry conditions recorded. This placed us in a position to identify propagation factors and to give recommendations for containment. For animals from private breedings, we detected *T. benhamiae* with a prevalence of 55.4%, which is a reduction of nearly 40% compared with animals from large breeding farms. As risk factors, we identified the type of husbandry and the contact to other breedings. Furthermore, certain animal races, like Rex guinea pigs and races with long hair in combination with curls were predestined for colonization with *T. benhamiae* due to their phenotypic coat characteristics. A prevalence for infections with *T. benhamiae* of 36.2% has been determined for symptomatic pet guinea pigs suspected of having dermatophytosis and is comparable to the study of Kraemer et al. showing a prevalence of 34.9% in 2009 in Germany. The prevalence in humans is stable with about 2–3% comparing the data of 2010–2013 and 2018 in Thuringia. The new type of *T. benhamiae* was by far the most frequent cause in all settings.

## 1. Introduction

Since the turn of the millennium, infections with the yellow phenotype of *T. benhamiae* have increased [1]. This fungus is not only isolated from symptomatic guinea pigs, but also causes highly inflammatory infections in humans, especially children, who have had contact with these animals. Kraemer et al. reported that in approximately one quarter of the cases, humans showed clinical signs of dermatophytosis. In half the households, only children were affected [2]. In the past it was unclear where the source of the fungal infection or colonization of the guinea pigs was located. Therefore, we had systematically examined guinea pigs in pet shops for the first time [1]. Up to now, there have been only single case reports of infections with *T. benhamiae* among staff of pet shops. Simultaneously with ours, a Dutch study [3] was published, which showed that 16.8% of the guinea pigs from pet shops carried dermatophytes, 88% of which were *T. benhamiae*. In contrast, our study was able to show that more than 90% of the 59 animals examined were mainly asymptomatically colonized, that is, they carry and can transmit the pathogen. Most of the animals in the 16 pet shops in Berlin were delivered by large commercial breeders (pers. comm./animal trader). The five animals from the only private breeding were free of *T. benhamiae*. This result allowed the assumption that animals from private breeders are generally less strongly colonized with the pathogen. Therefore, guinea pigs were sampled in 21 private breeders’ farms spread over Germany, and their husbandry conditions were documented. The study also served to collect data on the nationwide prevalence of *T. benhamiae* infections in humans and pet guinea pigs, as there are only limited studies available on this topic.

## 2. Material and Methods

### 2.1. Study Structure

#### 2.1.1. Private Breeding

To determine the prevalence of *T. benhamiae* in breedings, 21 private farms, spread over the whole country, were visited and 381 guinea pigs, symptomatic and asymptomatic, were sampled. Besides existing symptoms indicating a possible dermatophytosis, further data on the animal and the husbandry were documented to identify possible risk factors which favor a colonization with *T. benhamiae*. These factors included the type of housing environment and population dynamics, measurement of prevailing humidity, race and age of the animal, as well as different coat morphologies, such as hair length and the presence of curls and other coat-free areas.

#### 2.1.2. Pets

In order to analyze the frequency of *T. benhamiae* in skin infections of guinea pigs in German households, we received isolates from LABOKLIN (Bad Kissingen, Germany) and SYNLAB Vet (Augsburg, Germany) over a period of 10 months, from February to November 2019, which tested positive for dermatophytes in routine diagnostics with in-house PCR methods. In our laboratory, the exact species differentiation was then carried out. In total, we received 435 isolates, of which 236 isolates (192 cultures, 44 DNA isolates) were from LABOKLIN and 199 isolates (DNA isolates only) from SYNLAB Vet. Residual samples of the DNA isolates were stored at −20 °C for later analysis. Culture plates were stored at 4 °C.

### 2.2. Questionnaires

In order to determine the prevalence of *T. benhamiae* in humans and animals, questionnaires were sent to practicing veterinarians and dermatologists throughout Germany to obtain the number of cases for the year 2018.

About 900 dermatological practices and clinics as well as about 200 veterinary dermatologists were contacted directly via e-mail. A total of 3500 veterinarians were informed via the newsletter of the German Veterinary Medical Society (DVG). In addition, an advertisement with a link to the questionnaire was published in the monthly members’ magazine of the Bundesverband Praktizierender Tierärzte e.V. as well as in an issue of the Deutsches Tierärzteblatt. For the year 2018, the total number of examined patients and infections with *T. benhamiae* were questioned. For the *T. benhamiae* diagnoses, it was asked whether these were determined by molecular biological or cultural diagnostics. For cultural findings, the color of the culture could be additionally indicated. Dermatologists had the additional possibility to indicate the location of the human infection and whether guinea pigs were kept in the patient’s household (for the questionnaire, see Supplementary Materials). Veterinarians should additionally indicate the number of *T. mentagrophytes* infections in guinea pigs and the number of cases in which a zoonotic transmission to the keeper was known. If other dermatophytes have been detected on guinea pigs, the species and number could be noted additionally.

### 2.3. Diagnostics

#### 2.3.1. Sampling of Guinea Pigs

For the sampling, the animals were brushed intensively with a toothbrush according to MacKenzie, concentrated mainly on the back (dorsal), flanks (lateral), and head area. The use of interchangeable toothbrushes (yaweco, Bernau im Schwarzwald, Germany, medium, cat# 101020) allowed a quick transfer of the head brushes into a sterile 50 mL reaction tube (Falcon/Corning Science, New York, NY, USA).

#### 2.3.2. Culture

Clinical samples were cultivated on two different culture media, on Sabouraud agar with gentamicin and chloramphenicol and on Sabouraud agar with gentamicin, chloramphenicol, and cyclohexamide. Cultivation was at 28 °C for at least 3 weeks. If no growth was visible after this time, the sample was considered negative.

#### 2.3.3. DNA Extraction

For the subsequent DNA isolation from clinical samples, the Qiagen Blood & Tissue Kit (Qiagen, Hilden, Germany, cat# 69506) was used according to the manufacturer’s protocol with slight modifications. The brush was vortexed with 2 mL 0.9% sterile NaCl solution for 10 s. The NaCl solution was then transferred to a 2 mL reaction tube and centrifuged for 5 min at 10,000× *g*. The resulting pellet was resuspended in 230 µL ATL buffer (Qiagen, Hilden, Germany, cat# 19076) and, after the addition of 20 µL protein kinase K (Qiagen, Hilden, Germany, cat# 19131), was digested overnight at 56 °C instead of for 1 h. The DNA was eluted in 45 µL dH_2_O only.

Cultures: A single fungal colony was taken from grown culture and added to 60 µL lysis buffer (QuickExtract™ Plant DNA Extraction Solution, Lucigen, Middleton, Wisconsin, USA, cat# QEP 70750). An incubation of 1 h at 65 °C followed. After the lysis was stopped at 98 °C for 2 min, the lysate was used directly in the PCR.

### 2.4. PCR Method

To differentiate between variants of *T. behamiae*, a sequence region in the *ITS* region was chosen. The following primer sequences (5′3′) were used:

yellow variant: 5′-CGATAGGAATCAACGTTCCATC/5′-CCCCGAAAGAGGAGGT (for/rev)
white variant: 5′-GATAGGGACCAACGTTCCG/5′-CCCGAAAGAGGGGGC (for/rev)

The specificity of the primer pair was checked using NCBI blast option and DNA of close-related zoophilic species, for example, *T. erinacei* and *T. verrucosum*.

For the master mix of both PCR reactions, 7.4 µL dH_2_O, 0.8 µL each of the forward and reverse primer (50 pmol), and 10 µL Biozym Red HS Taq Master Mix (Biozym Scientific, Hessisch Oldendorf, Germany, cat# 331126) were used for each reaction (20 µL volume). The PCR started with an initial 3 min denaturation at 95 °C, followed by 40 cycles consisting of 15 s denaturation (95 °C), 15 s annealing (58 °C), and 20 s elongation (72 °C). The final elongation step was 60 s.

If neither of the two variants was detected, a pan-dermatophyte-specific PCR and external sequencing (LGC Genomics GmbH, Berlin, Germany) followed for species differentiation. For this PCR, the *ITS5* primer [4] was used in combination with a pan-dermatophyte-specific reverse primer [5] to amplify the entire *ITS1* region. The annealing temperature was 56 °C. If it was not possible to identify the species by sequencing, due to the presence of more than one dermatophyte species, a commercial test system (EUROArray Dermatomycosis, EUROIMMUN, Lübeck, Germany, cat# MN 2850-1005) was used.

### 2.5. Statistics

The statistical analysis was performed on SPSS (IBM SPSS Statistics 23). Ratio-scaled characteristics, such as the age of the animals, were tested for significance using Mann–Whitney U-test, and nominally scaled characteristics were tested for significance using chi-square tests. A *p*-value of <0.05 was considered significant.

Participation in the study was voluntary, free of charge, and all data were anonymized prior to analysis.

## 3. Results

### 3.1. Prevalence of Farm Animals

**Figure 1:**
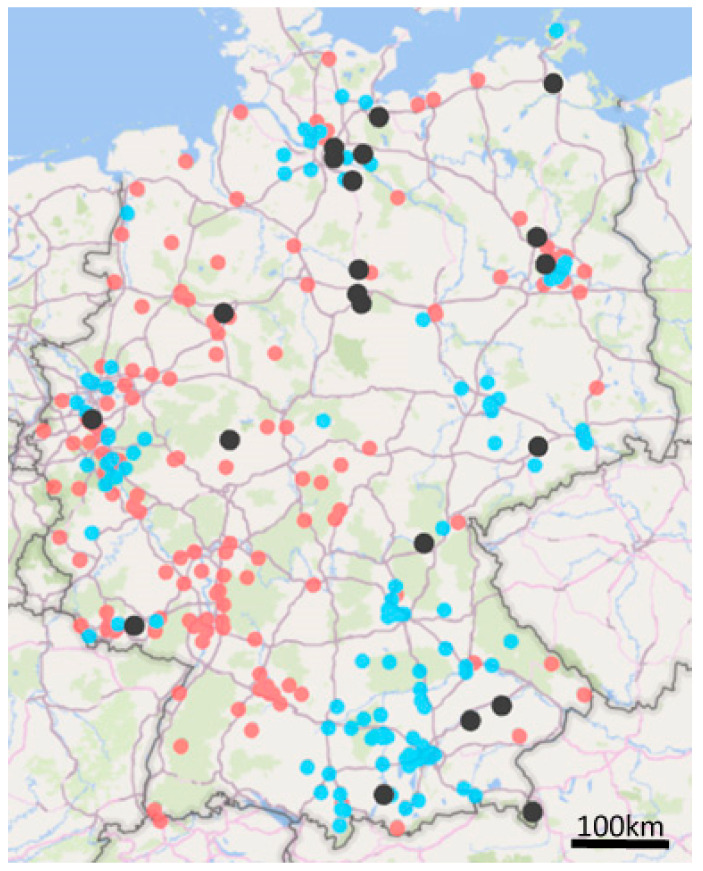
Sample origin, black—private breedings, blue—SYNLAB Vet, red—LABOKLIN.

**Figure 2:**
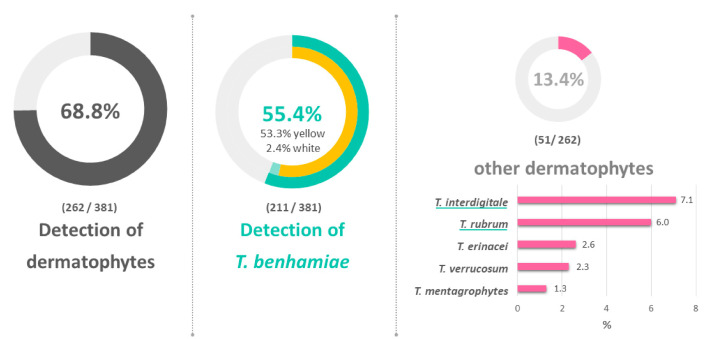
Detected dermatophytes on breeding guinea pigs.

Nationwide (except for Baden-Württemberg, Thuringia, and Saarland), 21 breedings with a total of 381 guinea pigs were sampled (Figure 1). The herd sizes varied between 7 and 100 animals. Samples were taken from twenty animals per breed. In herds with less than 20 animals, all of them were sampled. On 262 (68.8%) of the sampled animals, a dermatophyte was detected (Figure 2). With 55.4%, *T. benhamiae* was significantly the most frequently detected dermatophyte (*p* = 0.000325, Figure 2). No conclusions could be drawn either on the geographical origin or on differences in the geographical spread of *T. benhamiae*. In our survey, *T. benhamiae* was constantly distributed over the German territory. No regional hotspot and no index case were identified.

In 51 animals (13.4%) of other species, mostly anthropophilic dermatophytes such as *T. interdigitale* and *T. rubrum* were detected (Figure 2, Table 1). In 28 animals (7.3%), symptoms of dermatophytosis were documented. In all these cases, the yellow phenotype of *T. benhamiae* was isolated. The majority of the animals, 92.7%, were asymptomatically colonized, that is, carriers of the pathogens. On nine animals (2.4%), the white phenotype of *T. benhamiae* was detected. Fourteen mixed infections were observed. The most frequent were the combinations *T. interdigitale* and *T. rubrum* (6/14) or *T. interdigitale* and *T. verrucosum* (4/14).

### 3.2. Influence of Risk Factors

In order to identify risk factors that promote colonization or infection with *T. benhamiae*, possible factors related to the animal (sex and race) and the husbandry conditions (population dynamics and location) were documented during sampling (Table 2).

**Figure 3:**
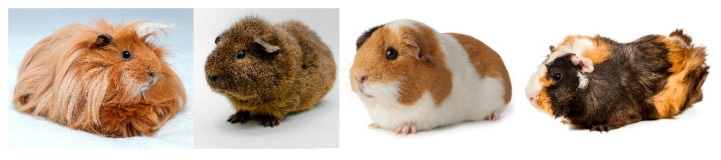
Guinea pig races with their different fur forms: (from left to right) longhaired guinea pigs, Rex guinea pigs, smooth-haired guinea pigs, curly-coated guinea pig (shutterstock.com/free-license images).

Castrated males were slightly more strongly colonized than uncastrated males (66.7% vs. 49.4%). If no differentiation was made between males, no significant differences (*p* = 0.506) between the sexes were found. In advance, different races were classified on the basis of morphological aspects. In longhair races, only the presence/absence of curls was distinguished. In the short-haired races, a distinction was made between Rex, Teddy, Rosette, and smooth-haired guinea pigs (Figure 3). Significantly (*p* = 0.00002) more frequently, longhaired races with curls (74.0%) and Rex guinea pigs (68.1%) were colonized with *T. benhamiae*. Smooth-haired guinea pigs were among the least affected. The influence of hairy curls, that is, the presence of fur-free areas due to a circular growth direction, was again examined in detail. Animals that were carriers of one or more curls showed significantly (*p* = 0.000328) more frequent colonization with *T. benhamiae* than animals without this feature (68.6% vs. 49.4%). If only short and long fur was differentiated, no significant difference was found. The husbandry system was differentiated between indoor husbandry (closed, heatable rooms, 22 °C on average, recorded relative humidity 58–66%), outdoor husbandry (open barn/open-air enclosure, temperature and relative humidity depending on weather) and cold barn management (open barn but protected from wind and weather, temperature and relative humidity depending on weather). Animals living in cold housing were the least affected (46.6%). Indoor animals were significantly (*p* = 0.011) more affected (76.2%). Finally, a classification of the breeds was made based on their stock dynamics. Dynamic breeding differs from static breeding by frequent new arrivals and departures. With the latter, the animal population is only subject to a few changes. These work with little contact to animals from other breeds. Animals from dynamic breeding were significantly (*p* = 1.19 × 10^−14^) more strongly infected with *T. benhamiae* than animals from autarkic breeding (76.0% vs. 37.1%).

Breeders and owners reported very often symptomatic infections on young animals at the age of 1 to 3 months, as well as on very old animals (>5 years). Neither the sampled breeding animals nor the data from symptomatic pets showed a significant influence of age on the prevalence of *T. benhamiae* (Mann–Whitney test *p* = 0.934). The age distribution (as average and median) of all animals and of the mostly asymptomatic breeding and symptomatic pets shows no relevant differences (Table 3). Only the median for symptomatic pets indicates that mainly younger guinea pigs were infected with the pathogen.

### 3.3. Prevalence of Symptomatic Pets

To investigate the incidence of dermatophytosis caused by *T. benhamiae* in guinea pigs, which are usually kept in pairs in the household, we received residual clinical samples with suspected infection from two large diagnostic laboratories, SYNLAB VET and LABOKLIN. With the support of the two large laboratories, we were able to cover samples from a wide area of Germany (Figure 1). Over a period of 10 months, February to November 2019, we differentiated 382 samples at species level. In total, the two laboratories received 9636 samples from guinea pigs during this period, of which 1035 (10.7%) were submitted with suspected dermatophytosis (Table 4). Of the 382 dermatophyte infections diagnosed, 375 (98.2%) were caused by *T. benhamiae*. In seven cases, other dermatophyte species were identified (Table 4). Using the PCR developed in this study, 357 (95.2%) *T. benhamiae* isolates were differentiated as yellow and 18 (4.8%) as white phenotype. Relative to the number of all guinea pig isolates examined in the two laboratories during this period, the prevalence of infection with *T. benhamiae* was 3.9%. From the suspected cases of dermatophytosis, 36.2% of the animals showed an infection with *T. benhamiae*.

### 3.4. Prevalence of Human T. benhamiae Infections

In order to record the prevalence of *T. benhamiae* infections in humans, questionnaires were sent to dermatological practices and clinics all over Germany and microbiological laboratories were contacted. A total of 51,238 patients could be registered who presented themselves in 13 dermatological practices in 2018 or whose samples were analyzed in 3 microbiological laboratories (Table 5). Using culture or molecular biological methods, in total 163 infections with *T. benhamiae* were detected, resulting in a prevalence of 0.3%. Among the 16 participants was the laboratory for medical microbiology in Mölbis (district of Leipzig), which has expertise in the diagnosis of skin fungal infections. Of the 163 infections with *T. benhamiae*, 124 were diagnosed with PCR methods in this laboratory alone, and 6044 patients were examined there during this period. The prevalence calculated from these data is 2.05% and thus about seven times higher compared with all laboratories (0.3%). Animal contact is described in 39 (31.5%) of the 124 patients who tested positive, and in 32 (25.8%) of them, specifically with a guinea pig.

## 4. Discussion

The massive spread of *T. benhamiae* in the last two decades is related to the parallel emergence of a new phenotype, which is characterized by a yellow instead of a white-colored culture [6]. Our study shows that in fact the white phenotype was only isolated from 2.4% and 4.8% of breeding guinea pigs and pets, respectively.

Furthermore, our study investigated for the first time the distribution of *T. benhamiae* in private breedings. With 55.4%, the prevalence of the pathogen was significantly lower than in animals from commercial animal wholesale. In a study in 2016, we were able to detect *T. benhamiae* on guinea pigs in Berlin pet shops, which usually came from non-animal welfare-compliant large breedings, with a prevalence of 93% [1]. In contrast, a study by Dutch colleagues at the same time was able to show that only 16.8% of guinea pigs from pet shops carried a dermatophyte, mostly *T. benhamiae*; however, no information on the origin of the animals was provided [3]. The same applies to a study from France in 2016, in which *T. benhamiae* was detected on 34 of the 51 examined guinea pigs (67%) in three pet shops in Nancy [7]. In a Danish study, *T. benhamiae* was detected on the guinea pigs offered in 41% of the pet shops investigated in Copenhagen. In this case, contaminated pet shops belonged exclusively to the group of shops that purchased their animals from external sources (animal traders) and had not bred the animals themselves [8]. For these three studies, in contrast to our study, the culture was used as an initial diagnostic procedure, which is known to have only about 70% of the sensitivity of PCR [9]. 

Poor hygienic conditions and exposition to carriers are known to promote the transmission and spread of the pathogen in the animal stock and thus have a negative effect on the constitution of the animals [10]. This leads increasingly to dermatophytosis. The probability of acquiring an infected animal is approximately 50% lower in private breeding than in a pet shop that purchases animals from animal wholesalers.

Nevertheless, even a husbandry which is oriented towards the needs of the animal seems not to be able to prevent an infection of the stock with *T. benhamiae*.

The aim of the study was also to identify risk factors that influence colonization. Animals from purely indoor housing systems were significantly more likely to be colonized with the pathogen than those from outdoor and cold housing systems. Reasons for this may be the better air exchange and the associated, weather-dependent, lower air humidity.

In addition, breedings that were subject to high stock dynamics, that is, which had many new arrivals from other husbandry systems, were particularly exposed to high levels of contamination. The contact to other breeds, be it through exchange of breeding animals or at meetings (e.g., at breeding shows), could be identified as a further factor for the introduction and spread of *T. benhamiae*. Future strategies for containment and prevention must also start here.

Not only the housing conditions but also intrinsic factors of the guinea pigs influenced the probability of acquiring an infection. Guinea pigs with smooth hairs and with very long fur without curls were slightly less exposed to the pathogen than those with hairs that were not attached but standing up. In the former, the coat morphology means that the skin surface is rarely exposed, that is, better protected from damaging influences (trauma, lesions, UV light). In the latter, the so-called Rex and Teddy guinea pigs, the coat morphology contributes to the often dry, scaly skin (pers. comm./breeders). The influence on races with partially unprotected skin is supported by the significantly more frequent detection of the pathogen in animals with curls in our study.

A significant influence of gender on the colonization with *T. benhamiae* was not determined in our study either. This result is consistent with other studies [10,11]. Bucks (unneutered males) usually sit alone or together with a neuter and have contact with females only for mating. This reduces the risk of infection, which would explain the slightly reduced stress on these animals. Castrated males usually sit together with females in larger groups. Here, our data show a slightly increased burden. However, this effect is cancelled out if we do not differentiate between bucks and castrates in males.

There was no significant influence of age on infection/colonization with *T. benhamiae* in our study, confirming the study of Drouot et al. [11]. However, the median age of symptomatic pets was 6 months lower than the median of all animals (6 instead of 12 months). Kraemer et al. [12] and Vangeel et al. [13] reported that affected animals were significantly younger than those with negative culture results as well as the healthy animals of a control population. In 2012, the group around Kraemer et al. [12] mentioned that 40% of infected guinea pigs were younger than 5 months and calculated a median of 6 months for their cohort. This is consistent with the observations of breeders and veterinarians, who increasingly report symptomatic mycoses, especially in guinea pigs in the first 3 months of life, which are suspected to be associated with an immune deficiency after weaning (4–6 weeks of life) (pers. comm./breeders, veterinarians). The observed shift in the median of symptomatic animals may be an indication.

Besides *T. benhamiae*, other dermatophytes were detected in 13.4% of the examined animals, mainly two anthropophilic species, *T. interdigitale* and *T. rubrum*, whose origin was most likely the breeders themselves. Kraemer et al. [12] report further dermatophytes isolated in guinea pigs such as *M. canis*, *M. audouinii,* and *T. rubrum*. In addition, other studies isolated *M. canis* in small numbers in Germany [14] and New Zealand [15]. These results, consistent with the present study, show that dermatophytes other than the main pathogen are only infrequently isolated from guinea pigs.

For symptomatic pet guinea pigs suspected of having dermatophytosis, we determined a prevalence of 36.2% for *T. benhamiae*. Kraemer et al. [12] reported a prevalence of 34.9% for *T. mentagrophytes* (old nomenclature) in a comparable survey for the year 2009. The authors noted that a morphological differentiation within the *T. benhamiae* species complex was not possible, but assumed that “the *Arthroderma* (*A.*) *benhamiae* complex is associated with most dermatophytoses in Guinea pigs and rabbits” [12]. Considering the very low prevalence of *T. mentagrophytes* of 1.3% for asymptomatic and 0.3% for symptomatic guinea pigs in our study, it is assumed that the majority in Kraemer`s study was due to *T. benhamiae*. Based on this assumption, there was only a slight increase (1.3%) of the prevalence within 10 years in Germany. An Italian study reported a lower prevalence of 24.7% in pet guinea pigs in 2015 (23/93) [16]. In 95.2% of the cases, the yellow type of *T. benhamiae* was isolated.

For human infections caused by *T. benhamiae,* we computed a prevalence of only 0.3% from the results of the questionnaires we sent out. However, we assume that the number of unreported cases is significantly higher due to problems with the species identification, which is difficult, as *T. benhamiae* was described under different names during the last decades, like *Arthroderma benhamiae* as teleomorph of *T. mentagrophytes* [17] or *T. mentagrophytes* var. *procellae* [18]. This assumption is supported by the data provided by the Laboratory for Medical Microbiology in Mölbis/Leipzig during the present study, revealing a prevalence of *T. benhamiae* in 2018 of 2.05%. The same laboratory published a similar prevalence of 2.9% between March 2010 and March 2013 [6]. The authors concluded that *T. benhamiae* is currently more common than *M. canis*, at least in some regions of Germany. This trend seems to continue. In Strasbourg, a similar trend was observed: *T. benhamiae* was temporarily more frequent than *M. canis* in at least three years (2011, 2012, 2014) of the 9 year survey [19]. These authors were also able to establish a connection to a pet guinea pig in the household in 75% of the *T. benhamiae* cases, of which, however, only 25.9% showed symptoms of dermatophytosis. Silent carriers of *T. benhamiae* represented a major part (86.7%) of the animals in our study, too.

## 5. Concluding Remarks

The zoonotic potential of guinea pigs for transmitting a dermatophyte should not be underestimated. Therefore, we recommend buying a guinea pig from private breeding rather than from wholesalers because the probability of acquiring an infected animal is approximately 50% lower. In any case, diagnostic screening of guinea pigs ideally by molecular methods is recommended. The transmission of *T. benhamiae* can be reduced by outdoor and cold housing systems, static populations, and intrinsic factors like the coat morphology (smooth hair, no curls). In the last 10 years in Germany, the frequency of *T. benhamiae* infections in pet guinea pigs did not change. In Thuringia, a part of Germany, however, the frequency of human *T. benhamiae* infections is stable but on a high level compared with 2010/13. Breeders and animal owners must therefore be made aware of the zoonotic risk and how to minimize it. Therefore, we have drawn up a guideline (see Supplementary Materials).

## Figures and Tables

**Table 1:** Detection of dermatophytes on breeding guinea pigs (*^1^—both types were present in one animal/*^2^—partly mixed infections).

Species	Number of Samples (Percent)
Negative	119/381 (31.2%)
Dermatophytes positive	262/381 (68.8%)
*T. benhamiae* (yellow) *^1^	203/381 (53.3%)
*T. benhamiae* (white)	9/381 (2.4%)
Other Dermatophytes *^2^	51/262 (13.4%)
*T. interdigitale*	27/262 (7.1%)
*T. rubrum*	23/262 (6.0%)
*T. erinacei*	10/262 (2.6%)
*T. verrucosum/eriotrephon*	6/262 (2.3%)
*T. mentagrophytes*	5/262 (1.3%)

**Table 2:** Description of possible risk factors, their observed frequency, and their influence on the detection of *T. benhamiae* (SH—shorthair). * Comparison between all animal races.

Factor	Characteristics	Frequency (*n*) (%)	*T. benhamiae* Detection (%)	*p*-Value
Sex	Female	259 (20.2)	55.2	0.074
	Male	77 (68.0)	49.4	
	Castrated	45 (11.8)	66.7	
	Male + castrated	122 (32.0)	55.7	0.506
Animal race/Pheno type	Long hair + curls	50 (13.1)	74.0	0.00002 *
	Long hair − curls	39 (10.2)	30.8	
	Teddy	90 (23.6)	51.1	
	Rex	69 (18.1)	68.1	
	Rosette (SH + curls)	69 (18.1)	62.3	
	Smooth hair	64 (16.8)	40.6	
Husbandry condition	Outdoor	114 (29.9)	51.8	0.011
	Cold barn	133 (34.9)	46.6	
	Indoor	134 (35.2)	67.2	
Stock Dynamics	Static (autarkic)	202 (53.0)	37.1	1.19 × 10^−14^
	Dynamic	179 (47.0)	76.0	
Coat length	short	300 (78.7)	56.3	0.276
	long	81 (21.3)	51.9	
Curls	Ye	118 (31.0)	68.6	0.000328
	No	263 (69.0)	49.4	

**Table 3:** Age distribution among the sampled animals.

Age	Average (Months)	Median (Months)
of all animals (breedings)	15.8	12
of animals positive for *T. benhamiae* (breedings) *	15.7	12
of symptomatic pets (laboratory data)	17.5	6

* Asymptotic significance (Mann–Whitney test) = 0.934 not significant.

**Table 4:** Detection of dermatophytes in guinea pig samples from SYNLAB VET and LABOKLIN, February–November 2019.

Samples Analyzed	Number
Total	9636
with suspected mycosis	1035
Tested positive for dermatophytes	382
*T. benhamiae*	375
*T. benhamiae* (yellow)	357
*T. benhamiae* (white)	18
*T. rubrum*	3
*Microsporum (M.) canis*	1
*T. mentagrophytes*	1
*T. spp.*	1
*T. interdigitale*	1

**Table 5:** Evaluation of questionnaires on recorded human *T. benhamiae* infections, 2018.

Patients Recorded	Number
Total	51,238
Number of *T. benhamiae* infections	163
of which culture diagnosed	126
thereof diagnosed by PCR	145
thereof contact with guinea pigs known	32/163

## Supplementary Materials

The following are available online at https://www.mdpi.com/2309-608X/6/3/161/s1. S1 Questionnaire sent to dermatologists and microbiological labs. S2 Guideline for owners and breeders of guinea pigs.
