# Supplementary material for: German-Wide Analysis of the Prevalence and the Propagation Factors of the Zoonotic Dermatophyte Trichophyton benhamiae
Source: J Fungi (Basel). 2020 Sep 3;6(3):161. doi: 10.3390/jof6030161 (PMC7558194; doi:10.3390/jof6030161)
Supplement: Supplementary file 1 [file jof-06-00161-s001.pdf]

Questionnaire on *Trichophyton benhamiae*

**Total number of patients in 2018**	
**Number of *T. benhamiae*-Infections 2018**	
• as Tinea capitis	
- davon Tinea capitis profunda	
• as Tinea faciei	
• as Tinea corporis	
• as Tinea corporis + Tinea capitis	
• as Tinea corporis + Tinea faciei	
• as Tinea manuum	
• as Tinea	
• thereof culture grown	

	creme	red	yellow/orange
- thereof cultural underside	/	/	/

• thereof diagnosed by PCR	
• thereof contact with guinea pigs known	
• thereof contact with animals known	
• thereof contact with animals unknown	
