# Supplementary material for: German-Wide Analysis of the Prevalence and the Propagation Factors of the Zoonotic Dermatophyte Trichophyton benhamiae
Source: J Fungi (Basel). 2020 Sep 3;6(3):161. doi: 10.3390/jof6030161 (PMC7558194; doi:10.3390/jof6030161)
Supplement: Supplementary file 2 [file jof-06-00161-s002.pdf]

GUIDELINE

Dermatopyhtosis of the guinea pig

Autors: Max Berlin and Yvonne Gräser – Nationales Konsiliarlabor für Dermatophyten am Institut für Mikrobiologie und Infektionsimmunologie, Charité – Universitätsmedizin Berlin Hindenburgdamm 27, 12203 Berlin

1. Introduction

Dermatophytes

Dermatophytes are filamentous fungi that use keratin as a carbon source, which is responsible for the form and function of skin, hair and horn or nails. We distinguish between zoophilic dermatophytes, which have a main animal host, and anthropophilic species, which are mainly isolated from humans. If a zoophilic dermatophyte is transmitted to humans, it is called a zoonosis. Until approx. 2013, *Microsporum* (*M.*) *canis*, mainly found on cats, was considered the most common zoonotic dermatophyte (Uhrlaß, 2015). After that it was replaced by *Trichophyton* (*T.*) *benhamiae* (formerly known as *T. mentagrophytes* or *Arthroderma benhamiae*) (Uhrlaß, 2015). Zoophilic dermatophytes cause highly inflammatory infections in humans with an intact immune system, as they are not adapted and the human being is therefore a false host (Hiernickel, 2016).

T. benhamiae

T. benhamiae is mainly isolated from guinea pigs. Even if this dermatophyte is also occasionally detected on other animals, especially small mammals like rabbits, rats and hamsters. Current numbers about the distribution of *T. benhamiae* on the guinea pig draw the following picture.

In a study in 2016, *T. benhamiae* was isolated of more than 90% of (55 of 59) of the guinea pigs sampled in 15 pet shops in Berlin (Kupsch, 2017). These animals mainly came from large breedings. In a Germany-wide prevalence study, which followed in 2019 and in which no guinea pigs from pet shops were sampled, but in 21 private breeds, only in 55.4% of the (211 of 381) of the animals *T. benhamiae* was detected. In both studies 87% (183 of 211) were asymptomatic carriers (colonized with the fungus but without symptoms). The fact that animals from conventional large-scale breedings are more severely affected than those from private breedings was also shown in a Danish study (Tekin, 2019). In 7 of 16 pet shops *T. benhamiae* was isolated from the guinea pigs. Of these 7, 5 obtained their animals from the European animal wholesale. Two from private breeders. All 9 pet shops with unencumbered guinea pigs received their animals from private breedings or bred themselves. Thus, it can be concluded that the condition of husbandry in private breeds reduce the spread of *T. benhamiae*, but cannot prevent a colonization.

First described in 1967 (Ajello, 1967), *T. benhamiae* played only a minor role until the turn of the millennium. This changed in the following years with the appearance of a new morphotype of this dermatophyte (yellow phenotype in culture instead of white). After first human cases in Japan (Nakamura, 2003), infections were increasingly observed in Europe as well (Fumeaux, 2004). Various studies have shown not only the progressive spread of *T. benhamiae*, but also its potential as a serious zoonosis (Drouot, 2009; Nenoff, 2014) and confirmed the guinea pig as the predestined host (Bartosch, 2019; Uhrlaß, 2015). Recent data from large microbiological laboratories show that *T. benhamiae* is responsible for almost 90% of the dermatophytoses on guinea pigs (Krämer et al.; our study).

2. Diagnosis

In the light of a Wood's lamp, *T. benhamiae*, like some other *Trichophyton* species, is not detected. *T. benhamiae* can be bred by cultural breeding but is very difficult to differentiate from other species. Macroscopically it is confused with *M. canis* due to the yellowish pigmentation of the colony obverse. Microscopically it is difficult to distinguish T. benhamiae from *T. interdigitale*, because the strains tend not to express macroconidia. In addition, there is a problem of false negative results, since dermatophytes do not always grow in culture or are overgrown by other molds. It takes 3-4 weeks until the result of the morphological identification is available.

The National Consiliary Laboratory for Dermatophytes recommends molecular biological detection. This method is fast (2-3 days) 30% more sensitive than culture (Kupsch, 2016). This predestines the method also for the detection on asymptomatic carrier animals. Veterinary practices do not have the resources for molecular detection. However, there are large vet. microbiological laboratories that perform diagnostics reliable and fast (synlab.vet GmbH / LABOKLIN GmbH & Co. KG / IDEXX Vet Med Labor GmbH). For the sampling of symptomatic animals as well as for screening of asymptomatic animals, the brush technique according to McKenzie is the best choice (Kupsch, 2017). For this purpose, a new toothbrush (still in an intact package) or sterile cytobrush brush (can be used, brushing over the coat for 1-2 minutes and over lesions.

High quality laboratory-based diagnostics not only enables fast and correct treatment, also protects against unnecessary medication by avoiding false diagnoses. The clinical picture of dermatophytosis can be diverse and not always be clearly differentiated from other diseases. A possible concomitant infestation of mites, for example, can distract from an existing dermatophyte infection. The data collected in our study show that almost 50% of the suspected diagnoses are not confirmed by diagnostic results. This means, that in about 50% of the cases where the diagnosis is made by the clinical picture alone the laboratory analysis could protect against wrong and unnecessary treatment. On the other hand, it also supports the identification of asymptomatic carrier animals and thus reduces the transmission to other animals and their human contact persons.

3. Measures

Always treatment in combination with environmental disinfection!

The biggest challenge is the high number of asymptomatic carrier animals. These animals are responsible for transmission and distribution in the animal population. Therefore, it is important to identify and treat these animals, in combination with a sufficient disinfection of the affected stables. Only then, a sanitation can be successful.

Topical treatment

symptomatic and asymptomatic animals (according to ESCCAP, 2009; Hein, 2016)

Imaverol® (Elanco Deutschland GmbH)

(1:50 dilution to obtain a final concentration of 2mg/ml)

The animal is placed in a flat dish for treatment and the solution is applied with a sponge. It is extremely important that the active ingredient covers the skin completely! Contact with the eyes, as well as licking, should be avoided. Then the animal is wrapped in a towel to protect it from drafts and hypothermia while drying. The active ingredient is not washed out again.

Application: 2x / week at least for 4 weeks.

Systemic treatment (in combination with topical treatment)

symptomatic animals

Itrafungol® (Elanco Deutschland GmbH)

Dosage: 5 mg/kg (= 0.5 ml/kg and day Itrafungol®-Lsg.) Treatment interval: 7 days, 7 days break, then again 7 days treatment etc. Depending on the progress of cure, the treatment consists of at least 2 - 3 cycles (4 to 6 weeks) and ends with a negative diagnostic result.

Disinfection of the environment

Without sufficient disinfection of all materials in contact with the animals, treatment is not very effective. Spores and fungal fragments of dermatophytes are able to survive on surfaces for long periods of time (up to 1.5 years).

Guinea pigs are often kept in wooden stables or on wooden surfaces. However, this material has major disadvantages in respect to effective disinfection.

For the duration of the treatment the animals should move into an extra pen, whose surface is easy to clean. A large cardboard box could be burned for example afterwards.

For a lasting effect, it is recommended to disinfect twice before the animals are put back into the pen. If the animals sit in a cardboard box during treatment, it should be changed once a week. If the treatment takes place in a barn, it should be disinfected at least once a week.

The following agents are available for this purpose:

Imaverol® (Elanco Germany GmbH)

(1:50 dilution to obtain a final concentration of 2mg/ml)

Apply with pump spray bottle or sponge.

The reaction time depends on the absorbency of the material: 30 min e.g. for plastic, up to 3 h e.g. for wood.

Sodium hypochlorite, also known as "chlorine bleach”

Commercially available cleaners from the drugstore or pharmacy with a sodium hypochlorite (NaClO) concentration between 7-13% are recommended.

Exposure time only 2 - 5 minutes, or depending on the manufacturer's recommendation.

Chlorine bleach has the advantage of being very fast and effective. It is also inexpensive and disintegrate quickly into the harmless components sodium chloride, oxygen and water. Disadvantages are the odor, irritation of the mucosa and respiratory tract at higher concentrations. Therefore, good ventilation during disinfection and adequate skin protection must be ensured. Due to the bleaching effect, however, it is not suitable for all surfaces and clothing.

4. Risk factors and prevention

Immune insufficiency

The clinical manifestation of a dermatophyte infection is often the expression of an immune deficiency. Factors that can lead to a weakening of the immune system are

- Stress (e.g. transport and introduction into a new group)

- Weaning from the mother (4-6 weeks of life)

- Other diseases and parasite infestation

Other factors:

- Lactating guinea pigs can transfer existing dermatophytes to the young animals.

- Ectoparasites (fleas, ticks, mites, hairy parasites) and/or itching caused by other diseases are possible causes of microtrauma predisposing guinea pigs to dermatophytosis.

- Warm and humid climate increases the risk of dermatophytosis (indoor housing with poor ventilation). This type of climate favors the proliferation of the pathogens and an increased pathogen load increases the risk of infection.

Exhibitions

Exhibitions and awards carry a high risk of infection due to the massive contact with animals from other breedings. For a transmission no direct contact between the animals is necessary. Dermatophytes can also be transmitted via the breeder's hand or surfaces. It is recommended that event organisers identify these points of transmission and take subsequent measures.

Hygiene

After each contact with animals, wash hands thoroughly and all skin areas that have been in contact with the animal. For disinfection, commercially available skin disinfectants with a proven fungicidal effect are sufficient. Hair and skin particles adhere particularly well to clothing. A change of clothing is recommended when changing between animals in quarantine and the rest of the stock. For the disinfection of textiles a wash cycle at 60C is sufficient.

New additions /Quarantine

Newly acquired animals should first be put into quarantine. Transportation and moving to a new environment means stress, which can promote the clinical manifestation of dermtophytosis, especially in guinea pigs. In view of the high number of asymptomatic carriers, the absence of signs of infection cannot be considered an indication of the absence of the pathogen. Therefore, screenings of the animal and its surroundings is recommended (see also notes under diagnosis) and in case of a positive result, a spray treatment of the animals and surfaces with Imaverol (see local treatment). Finally, a therapy control should be performed before the animal is moved to the actual herd. Without prior screening, new arrivals should always be treated preventively, i.e. topical antifungals.

Vaccination

For guinea pigs there is no approved vaccination available yet. For cats, dogs and horses, there is a vaccination against a selection of the most common dermatophytes (Insol Dermatophyton®, Boehringer Ingelheim). However, it does not contain *T. benhamiae* nor is it able to prevent infection and dermatophytosis. Successful vaccination only increases the infectious dose necessary to trigger infection. Reduces, in the best case, only the risk of infection and can not guarantee 100% protection.

5. Appel

Dermatophytes must be taken seriously! The current situation requires an open approach to the topic, from the zoonosis aspect and in order to preserve animal welfare. Scientific surveys show the

wide spread of *T. benhamiae* on guinea pigs. With the right awareness of the issue among owners, breeders and veterinarians, there is a chance that the situation will be improved in the long term.

6. Summary

- *T. benhamiae* has spread ubiquitously to guinea pigs since the turn of the millennium and is an emerging problem for animals and humans.

- Between 50% and 90% of the animals are carriers without symptoms.

- The extent of the burden depends strongly on the origin of the animals. Animals from private breedings are clearly less often colonized than animals from wholesale.

- Measures are necessary to stop the spread or to achieve containment.

- For a sustainable success the following measures must be implemented:

1. fast and correct diagnosis.

2. effective therapy of all affected and confined animals.

3. hygiene (clean-out procedure, comprehensive cleaning and disinfection of the stables, hand disinfection, protective clothing)

7. References

Ajello L, Cheng S-L, "The perfect state of Trichophyton mentagrophytes." Sabouraudia (1967) 4:230-234

Bartosch T, Frank A, Günther C, et al. "Trichophyton benhamiae and T.mentagrophytes target guinea pigs in a mixed small animal stock" Medical Mycology Case Reports (2019) 23:37-42

Drouot S, Mignon B, Fratti M, et al. "Pets as the main source of two zoonotic species of the Trichophyton mentagrophytes complex in Switzerland, Arthroderma vanbreuseghemii and Arthroderma benhamiae." Veterinary dermatology (2009) 20.1:13-18.

ESCCAP, Bekämpfung von Dermatophyten bei Hunden und Katzen, Empfehlung Nr. 2, 2009

Fumeaux J, Mock M, Ninet B, et al. "First report of Arthroderma benhamiae in Switzerland." Dermatology (2004) 208(3):244-50

Hein J, „Dermatophytose bei Kleinsäugern – immer eine ernst zu nehmende Infektion!" kleintier konkret, Medizinverlage Stuttgart (2016) 02:35-39

Hein J, „Dermatophytose bei Kaninchen und Meerschweinchen – ein Update einer Zoonose" Kleintierpraxis (2016) 12:675–688

- Hiernickel C, Wiegand C, Schliemann S, et al. „Trichophyton Spezies von Arthroderma benhamiae Klinisch therapeutische Aspekte eines neuen Erregers in der Dermatologie" Hautarzt (2016) 67:706–711

Kraemer A, Hein J, Heusinger A, et al. "Clinical signs, therapy and zoonotic risk of pet guinea pigs with dermatophytosis. Mycoses (2013) 56:168–17

Kraemer A, Mueller RS, Werckenthin C, et al. "Dermatophytes in pet Guinea pigs and rabbits" Veterinary Microbiology (2012) 157:208–213

Kupsch C, Berlin M, Graeser Y, "Dermophytes and guinea pigs: An underestimated danger?" Hautarzt (2017) 68: 827.

Kupsch C, Ohst T, Pankewitz F, et al. "The agony of choice in dermatophyte diagnostics—performance of different molecular tests and culture in the detection of Trichophyton rubrum and Trichophyton interdigitale" Clinical Microbiology and Infection (2016) 735.e11-735.e17

Nakamura Y, Kano R, Nakamura E, et al. „Case Report. First report on human ringworm caused by Arthroderma benhamiae in Japan transmitted from a rabbit." Mycoses (2003) 45:129-131.

Nenoff P, Uhrlaß S, Krüger C, et al. "Trichophyton species of Arthroderma benhamiae - a new infectious agent in dermatology." J Dtsch Dermatol Ges J Ger Soc Dermatol JDDG (2014) 12:571-581.

Tekin HG, Sigsgaard V, Zachariae C, et al. „Would you like to purchase a rodent with dermatophytes?" Mycoses. (2019) 62: 584- 587.

Uhrlaß S, Krüger C, Nenoff P, "Microsporum canis: Current data on the prevalence of the zoophilic dermatophyte in central Germany." Hautarzt (2015) 66: 855.

Uhrlaß, Ebert A, Krüger C, „Trichophyton Spezies von Arthroderma benhamiae - ein neuer häufiger zoophiler Dermatophyt in Deutschland - Daten zur Prävalenz im mitteldeutschen Raum." Derm Prakt Dermatol (2013) 19:370–372.

Scientific consulting by:

Dr. Jutta Hein

Specialist veterinary surgeon for pets

Head of the DVG-DGK working group small mammals (www.dvg-kleinsaeuger.de)

www.heimtieraerztin.de

Disclaimer: The information in this recommendation is based on the experience and knowledge of the authors and has been checked for accuracy with the greatest possible care. However, the authors and publishers accept no liability for any consequences resulting from a misinterpretation of the information contained herein, and furthermore offer no guarantee of success.
